# The Upregulation of Molecules Related to Tumor Immune Escape in Human Pituitary Adenomas

**DOI:** 10.3389/fendo.2021.726448

**Published:** 2021-10-21

**Authors:** Zhiyu Xi, Pamela S. Jones, Masaaki Mikamoto, Xiaobin Jiang, Alexander T. Faje, Chuansheng Nie, Kathryn E. Labelle, Yunli Zhou, Karen K. Miller, Roy J. Soberman, Xun Zhang

**Affiliations:** ^1^ Neuroendocrine Unit, Massachusetts General Hospital and Harvard Medical School, Boston, MA, United States; ^2^ Department of Neurosurgery, Massachusetts General Hospital and Harvard Medical School, Boston, MA, United States; ^3^ Nephrology Division, Massachusetts General Hospital and Harvard Medical School, Boston, MA, United States

**Keywords:** pituitary adenoma, immune checkpoint blockade, immunotherapy, immune escape, aggressive pituitary adenoma

## Abstract

Human pituitary adenomas are one of the most common intracranial neoplasms. Although most of these tumors are benign and can be treated medically or by transsphenoidal surgery, a subset of these tumors are fast-growing, aggressive, recur, and remain a therapeutic dilemma. Because antibodies against immune checkpoint receptors PD-1 and CLTA-4 are now routinely used for cancer treatment, we quantified the expression of mRNA coding for PD-1, CLTA-4, and their ligands, PD-L1, PD-L2, CD80, and CD86 in human pituitary adenomas and normal pituitary glands, with the ultimate goal of exploiting immune checkpoint therapy in aggressive pituitary adenomas. Aggressive pituitary adenomas demonstrated an increased expression of PD-L2, CD80, and CD86 in compared to that of normal human pituitary glands. Furthermore, aggressive pituitary tumors demonstrated significantly higher levels of CD80 and CD86 compared to non-aggressive tumors. Our results establish a rationale for studying a potential role for immune checkpoint inhibition therapy in the treatment of pituitary adenomas.

## Introduction

Pituitary adenomas are the second most common primary intracranial tumor (1–3). While the majority of these benign tumors can be managed effectively medically or with transsphenoidal surgery, there is a subset that remains resistant and/or recurs (4, 5). While there are no reliable predictors of aggressive behavior, elevated Ki67 index >3%, tumor invasiveness, large size, functioning adenomas, silent adenomas, and the rare event of distant metastasis have all been correlated with increased tumor aggressiveness (6–8). For tumors that grow despite radiation therapy, the alkylating agent temozolomide is commonly used. Only 50% of patients respond, and the vast majority of these tumors escape control (9). Therefore, finding additional therapeutic modalities is critical to their management.

The host response to tumors involves the recruitment and activation of T cell subsets and macrophages. Tumors escape this response by expressing immune checkpoint molecules on their surface. These, in turn, interact with their ligand/receptor on the surface of immune cells down-regulating their anti-tumor function (10). Among the immune checkpoint pathways, the most well studied molecules are programmed cell death protein 1 (PD-1), cytotoxic T-lymphocyte-associated antigen 4 (CTLA-4) and their ligands (11–15). PD-1 is mostly located on the surface of activated T cells, B cells, monocytes, natural killer (NK) cells, and dendritic cells. It has two ligands: programmed death-ligand 1 (PD-L1) and programmed death-ligand 2 (PD-L2) (16, 17). Unlike PD-1, which inhibits immune activity after T cells are activated, CTLA-4, which is mainly expressed on regulatory T cells, regulates the activation of T cells (14, 15). The CTLA-4 ligands are CD80 and CD86, which are found on antigen presenting cells. Monoclonal antibodies that block PD-1, PD-L1, and CTLA-4 have each been found to demonstrate persuasive anti-tumor effects (18–21). Immune checkpoint therapy has been effective in treating selective tumors, as exemplified by melanomas and renal cell carcinoma (22, 23).

The large majority of immune checkpoint blockade applications have been in the treatment of malignant tumors (e.g. melanomas), and there are few data on their efficacy in the treatment of aggressive benign tumors. Some reports showed increased expression of PD-L1 in functioning pituitary adenomas (24, 25), and recently, Lin et al. (26) reported a case of refractory Cushing’s disease (CD) in which a dramatic response to ipilimumab (anti-CLTA-4 antibody) and nivolumab (anti-PD-1 antibody) was observed. This suggests that the use of immune checkpoint blockade agents may be effective in a subset of refractory cases. We therefore analyzed mRNA expression of immune checkpoint molecules including PD-1, PD-L1, PD-L2, CLTA-4, CD80, and CD86, in clinically aggressive pituitary adenomas, adenomas that had not exhibited aggressive behavior and normal pituitary tissue to explore whether there is a scientific rationale for investigating whether immunotherapeutic agents are effective against aggressive pituitary tumors.

## Materials and Methods

### Sample Collection

Fresh human pituitary samples were provided by the Massachusetts General Hospital Neurosurgery service. A total of 60 tumor samples were collected; among them, 43 samples were aggressive tumors (28 clinically non-functioning, 7 GH-secreting, 4 ACTH-secreting, 3 PRL-secreting adenomas, as well as 1 silent TSH-expressing) and 17 samples were typical benign adenomas (11 clinically non-functioning, 4 GH-secreting, 1 ACTH-secreting, and 1 PRL-secreting adenomas) that had exhibited no signs of aggressive behavior. Aggressive tumors were defined as adenomas that were recurrent despite surgery and radiation, giant adenomas (≥4cm) with invasiveness, adenomas requiring multiple surgeries and radiation therapy, macroadenomas that recurred unusually rapidly (typically within 2-3 years) requiring radiation, and invasive macroadenomas. One control group was macroadenomas that were non-invasive and not recurrent following surgery. In addition to the tumor controls, 12 normal pituitary tissue samples were obtained from Harvard Brain Bank. These data are locate in **Supplementary Table 1**. This research was approved by the Mass General Brigham institutional review board.

### Total RNA Extraction

Briefly, tissues were cut into pieces and homogenized in TRIZOL reagent (Invitrogen, Waltham, MA, USA; 1 mL TRIZOL reagent per 50~100 mg tissue). The samples were kept at room temperature for a few minutes, then centrifuged. The supernatant was transferred to a new tube, and 0.2 ml chloroform was added followed by vigorous vertexing. After one additional centrifugation, the upper aqueous phase was collected, and an equal volume of isopropyl alcohol was added. The mixture was incubated at room temperature for 10 minutes and centrifuged to precipitate the RNA. Finally, the RNA was washed and dissolved, then diluted for spectrophotometric analysis to determine the concentration.

### RT-qPCR

First strand cDNA was synthesized using iScript cDNA Synthesis Kits (Bio-Rad, Hercules, CA, USA), starting with 1 µg of mRNA and finishing with 50 µL in volume. Quantificational PCR was performed with PowerUp™ SYBR™ Green Master Mix (Applied Biosystems, Foster City, CA, USA) following the manufacturer’s instructions. Each PCR mix contained 1 µL of cDNA and 20 nmol of each primer; and each reaction started with 50 C for 2 min, followed by 95 C for 10 min and 40 cycles of 95 C for 15 sec followed by 60 C for 1 min. The primer sequences used were as follows:

PD-1: 5’- CCAGGATGGTTCTTAGACTCCC -3’(forward),
5’- TTTAGCACGAAGCTCTCCGAT -3’ (reverse);
PD-L1: 5’- TGGCATTTGCTGAACGCATTT -3’(forward),
5’- TGCAGCCAGGTCTAATTGTTTT -3’(reverse);
PD-L2: 5’- ATTGCAGCTTCACCAGATAGC -3’(forward),
5’- AAAGTTGCATTCCAGGGTCAC -3’(reverse);
CTLA-4: 5’- GCCCTGCACTCTCCTGTTTTT -3’(forward),
5’- GGTTGCCGCACAGACTTCA -3’ (reverse);
CD80: 5’- AAACTCGCATCTACTGGCAAA -3’(forward),
5’- GGTTCTTGTACTCGGGCCATA -3’(reverse);
CD86: 5’- CTGCTCATCTATACACGGTTACC -3’(forward),
5’- GGAAACGTCGTACAGTTCTGTG -3’(reverse);
GADPH: 5’- GGAGCGAGATCCCTCCAAAAT -3’ (forward),
5’- GGCTGTTGTCATACTTCTCATGG -3’ (reverse).

### Statistical Analysis

Data were analyzed using JMP Pro 15 (SAS, Cary, NC, USA). All data are expressed as median (interquartile range). Statistical comparisons were performed using Wilcoxon rank sum test. A two-tailed *p* < 0.05 indicates a statistically significant difference.

## Results

### Adenoma *vs.* Normal Pituitary Samples

**Figure 1:**
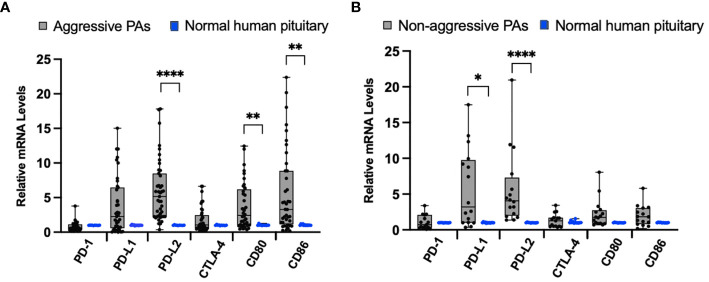
Immune checkpoint molecule mRNA expression in pituitary adenomas (PAs) compared to normal human pituitary tissue. **(A)** Comparison of relative mRNA levels of immune checkpoint molecules between aggressive PAs (gray bar, black dots) and normal human pituitary tissue (blue dots). **(B)** Comparison of relative mRNA levels of immune checkpoint molecules in non-aggressive PAs (gray bar, black dots) and normal human pituitary tissue samples (blue dots). *p < 0.05; **p < 0.01; ****p < 0.0001.

To examine the differences between aggressive behaving tumors (N=43) and normal pituitary samples (N=12), we compared the mRNA levels of the immune checkpoint molecules PD-1, PD-L1, PD-L2, CTLA-4, CD80, and CD86 by qRT-PCR. As shown in **Figure 1A**, there were significantly higher mRNA levels of PD-L2 (*p* < 0.0001), CD-80 (*p* = 0.0035), and CD-86 (*p* = 0.004) in aggressive pituitary adenomas. However, there was no significant difference in PD-1, PD-L1, and CTLA-4 mRNA levels between aggressive pituitary adenomas and normal pituitary samples.

When we compared the relative mRNA levels of the immune checkpoint molecules between non-aggressive tumors (N=17) and normal pituitary samples, we found significantly higher mRNA expression of ligand PD-L1 (*p*=0.02), PD-L2 (*p <* 0.0001) (**Figure 1B**). There was no significant difference in PD-1, CTLA-4, CD80, and CD86 mRNA expression between non-aggressive pituitary adenomas and normal pituitary samples.

### Aggressive *vs.* Non-Aggressive Pituitary Adenomas

**Figure 2:**
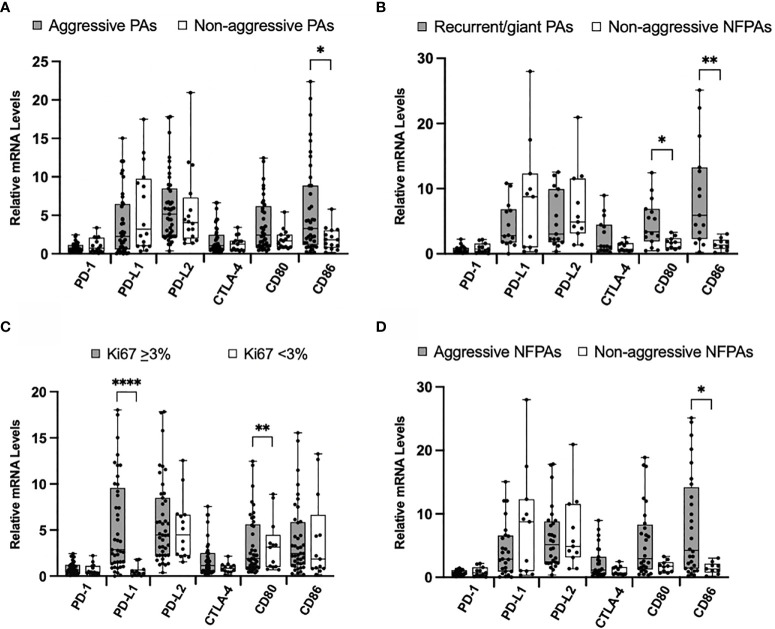
Immune checkpoint molecule mRNA expression in aggressive PAs compared to non-aggressive PAs. **(A)** Comparison of relative mRNA levels of immune checkpoint molecules between all aggressive PAs (gray) and all non-aggressive PAs (white). **(B)** Comparison of relative mRNA levels of immune checkpoint molecules between PAs recurrent through radiation or giant, invasive on presentation (gray) compared to non-functioning non-aggressive PAs (white). **(C)** Comparison of relative mRNA levels of immune checkpoint molecules between PAs with Ki67 ≥ 3% (gray) and PAs with Ki67 < 3% (white). **(D)** Comparison of relative mRNA levels of immune checkpoint molecules between non-functioning aggressive PAs (gray) and non-functioning non-aggressive PAs (white). *p < 0.05; **p < 0.01; ****p < 0.0001.

When we compared the levels of mRNA representing immune checkpoint molecules between these 43 aggressive tumors and 17 non-aggressive tumors (**Table 1**), only levels of CD86 were significantly higher in aggressive tumors (*p* = 0.035) (**Figure 2A**). No statistically significant differences were found between PD-1, PD-L1, PD-L2, CLTA-4, and CD80 mRNA levels in these groups.

**Table 1:** Clinical features of pituitary adenoma patients.

	All patients	Aggressive adenomas	Non-aggressive adenomas
N	60	43	17
**Age**	51.8	50.1	56.2
**Gender (male, %)**	38 (63.3%)	28 (65.1)	10 (58.8)
**Classification**			
** NFA**	39	28	11
** GH**	12	8	4
** ACTH**	5	4	1
** Prolactin**	4	3	1
**Tumor size (mean, cm)**	2.7	3.1	1.7
**Ki67 (n = 59, %)**			
** <3**	45	28	17
** ≥3**	14	14	0

NFA, nonfunctioning adenoma; GH, growth hormone-secreting adenoma; ACTH, adrenocorticotropic hormone-secreting adenomas.

We also compared a subset of aggressive tumors that were recurrent through radiation or were giant, invasive adenomas on presentation (N=15) to non-aggressive clinically non-functioning (N=11) pituitary adenomas. The mRNA levels of CD80 and CD86 were significantly higher in the aggressive tumors (*p* = 0.03, *p* = 0.002, respectively) (**Figure 2B**). No statistically significant difference was found for PD-1, PD-L1, PD-L2, and CLTA-4 mRNA levels between groups.

Using the well-established marker of Ki67 as an indicator of tumor aggressiveness, we compared mRNA levels of immune checkpoint molecules between tumors with Ki67 < 3.0% and those with Ki67 ≥ 3.0%. We found that the mRNA levels of PD-L1 were significantly elevated in tumors with higher Ki67 (*p* < 0.0001) (**Figure 2C**). No statistically significant difference was found for PD-1, PD-L2, CLTA-4, CD80, and CD86 mRNA levels between groups.

Finally, we compared the non-functioning aggressive (N=28) to the non-aggressive (N=11) pituitary adenomas. mRNA levels of CD86 were significantly higher in aggressive tumors (*p* = 0.02) (**Figure 2D**). No statistically significant difference was found for PD-1, PD-L1, PD-L2, CLTA-4 and CD80 mRNA levels between aggressive and non-aggressive NFTs.

### Functioning *vs.* Non-Functioning Adenoma Samples

**Figure 3:**
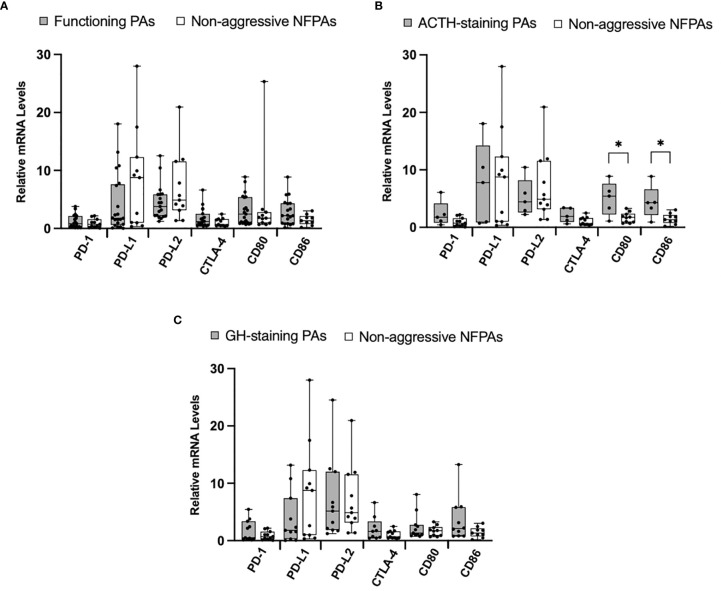
Immune checkpoint molecule mRNA expression in functioning PAs compared to non-functioning PAs. **(A)** Comparison of relative mRNA levels of immune checkpoint molecules between functioning PAs (gray) and non-functioning non-aggressive PAs (white). **(B)** Comparison of relative mRNA levels of immune checkpoint molecules between ACTH-staining PAs (gray) compared to non-functioning non-aggressive PAs (white). **(C)** Comparison of relative mRNA levels of immune checkpoint molecules between GH-staining PAs (gray) compared to non-functioning non-aggressive PAs (white). *p < 0.05.

Given reports suggesting higher immune checkpoint molecules in functioning tumors, we next explored the differences in our cohorts of functioning (N=21) and nonfunctioning non-aggressive tumors (N=11). We found no significant difference in relative mRNA levels of PD-1, CTLA-4, CD80, and CD86 (**Figure 3A**). We then compared ACTH-staining tumors (N=5) to nonfunctioning non-aggressive pituitary adenomas. The mRNA levels of CD80 and CD86 were significantly elevated in ACTH tumors (*p* = 0.02, *p* = 0.04, respectively) (**Figure 3B**). We also analyzed GH-staining tumors (N=12) compared to nonfunctioning non-aggressive pituitary adenomas. There were no statistically significant differences in mRNA levels between the groups (**Figure 3C**).

## Discussion

The last decade has seen a rapid growth in the use of immunotherapy, and specifically checkpoint blockade, to successfully treat a variety of solid tumors. While pituitary adenomas remain a largely benign disease, the challenging nature of invasive, recurrent, and/or hormonally functioning tumors leaves a gap in current management. In this study, we have found that the mRNA expression levels of the ligands for immune checkpoint receptors PD-1 and CTLA-4—namely, PD-L1 and PD-L2; CD80 and CD86, respectively— were all significantly higher in pituitary adenomas than in the normal human pituitary.

Furthermore, the mRNA levels of CD80 and CD86 were significantly higher in the most aggressive subset of PAs compared to non-aggressive pituitary adenomas. Tumors with Ki67 elevated to ≥3%, levels correlated with an increased risk of recurrence, had significantly elevated PD-L1 and, although we found no differences in immune checkpoint molecules among the functioning adenomas compared to non-functioning adenomas at large, a small subset of 4 ACTH-secreting tumors had significantly elevated CD80 and CD86.

The tumor cells and their surrounding components, including fibroblasts, immune cells, vascular networks, extracellular matrix, etc., comprise the tumor microenvironment (TME) (27). Physiologically, these stromal components maintain homeostasis, immune regulation and anti-tumorigenesis (28, 29). We found higher expression of PD-L2, CD80, and CD86 in aggressive pituitary adenomas when compared to normal pituitary tissues, suggesting the accumulation of peripheral immune cells like regulatory T cells, NK cells and dendritic cells in the pituitary tumor immune microenvironment (TIME). In non-aggressive pituitary adenomas, we found higher expression of PD-L1 and PD-L2 when compared to normal pituitary tissues. This indicates not only that increased immune infiltrate is not unique to aggressive tumors, but also that non-aggressive tumors infiltrates may be linked closer to PD-1 mediated pathways and aggressive tumors to CTLA-4 pathways. PD-1 mRNA, which is expressed by activated T cells, was found at the same level in tumor and normal tissues. These findings suggest that PD-1 and CTLA-4 immune checkpoint pathways may be useful therapeutic targets for pituitary adenomas.

Our transcriptional data complements recent findings of proteomic work by other groups by showing that, despite their benign nature, pituitary adenomas are not immunologically inert environments. In 2016, Mei et al. first reported that PD-L1 RNA and protein expression were significantly increased in functioning tumors compared to non-functioning adenomas (25). While this group and others have studied and found increased immune markers in functioning tumors, we focused on tumors with a clinically aggressive course, which included both functioning and non-functioning pituitary adenomas. In the most aggressive tumors, those that had recurred following surgery and radiation and those that were giant and invasive at presentation, we found significantly elevated CD80 and CD86. Furthermore, in tumors with elevated Ki67 index ≥3%, we found significantly elevated levels of PD-L1 mRNA levels. This is consistent with data from Wang et al., who reported that PD-L1 immunostaining occurred more frequently in tumors with Ki-67 index ≥3%, as well as increased PD-L1 immunostaining in GH and prolactin-secreting tumors (24). Our data demonstrated higher levels of PD-L1 mRNA in pituitary adenomas as compared to normal pituitary gland, but we did not find differences in immune markers when comparing all functioning pituitary adenomas to non-functioning pituitary adenomas. We did find that a subset of five ACTH-staining tumors had significantly elevated CD80 and CD86 mRNA levels compared to our nonfunctioning controls. Kemeny et al. found elevated PD-L1 immunostaining in human ACTH-secreting tumors and, in a murine model of Cushing’s disease, found that treatment with anti-PD-L1 led to restricted tumor growth and lower ACTH production (30). These findings are consistent with a potential role for checkpoint blockade in the management of pituitary adenomas with an aggressive clinical course, particularly refractory Cushing’s disease.

The significance of increased CD80 and CD86 expression in pituitary adenomas has not been previously reported, but we hypothesize it predicts an increased reliance on CTLA-4 mediated pathway of tumor suppression. Both CD80 and CD86 serve as co-stimulatory molecules in the immune environment, serving to activate T cells when they bind to CD28 or deactivate T cells when they bind to CTLA-4. These proteins are the most ubiquitous members of the B7 ligand family but are not currently targets for immune checkpoint blockade. B7-H3, another member of the B7 ligand family, is a target currently under active investigation for antibody-based immunotherapy, as it has been found to be expressed in many different cancer types but has a limited expression in normal tissues (31). Next steps may be to further study this ligand expression in pituitary adenomas. Furthermore, our data suggest that it is possible that CD80, CD86, and the CTLA-4-dependent tumor immune escape is involved in the development of tumor aggressiveness, a hypothesis that also merits further study.

Despite the success of immune checkpoint blockade, there remain significant challenges in predicting which patients will respond to therapy. Correlating treatment response with immune checkpoint expression remains an active area of investigation and some studies have shown that anti-PD-1/PD-L1 drugs lead to improved outcomes in patients harboring tumors with high PD-1/PD-L1 expression (32, 33). In addition, Van Allen et al. showed that patients who achieved clinical benefit from the anti-CTLA-4 drug ipilimumab for metastatic melanoma had higher levels of CTLA-4 and PD-L2 expression (34). Our results demonstrating increased expression of PD-L2, CD80, and CD86 in aggressive pituitary adenoma samples provide a rationale for studying whether immune checkpoint blockade is effective for tumor control. Lin et al. attributed the response of a corticotroph pituitary carcinoma to ipilimumab and nivolumab in part to the hypermutated status of the tumor (26). This group is leading a multi-center clinical trial (NCT04042753) to further investigate the efficacy of this combined immunotherapy.

There are several limitations of our work. First, the findings of our study are limited by these known challenges in correlating levels of immune checkpoint molecules with response. Furthermore, our data included small sample sizes, particularly in sub-groups of functioning adenomas. Given promising results in the role of immune checkpoint therapy for corticotroph adenomas, a larger analysis of ACTH-secreting tumors would be valuable.

Overall, our findings indicate that there is a significant immunologic profile difference between pituitary adenomas and normal pituitary as well as between clinically aggressive pituitary adenomas and non-aggressive pituitary adenomas. Our results suggest a possible role of immune checkpoint pathways in pituitary adenoma tumorigenesis and growth and also support a potential role for immune checkpoint blockade in pituitary adenomas that prove difficult to control with standard therapies. The increased expression of the ligands for PD-1 and CLTA-4 in human pituitary tumors suggests that immunotherapeutic antibodies such as ipilimumab and nivolumab may be able to directly target clinically aggressive pituitary tumors resistant to therapy. In addition, although we had small numbers of tumor samples on which to base any firm conclusions, our data suggest that corticotroph tumors may be particularly targetable by these agents.

## Data Availability Statement

The original contributions presented in the study are included in the article/**Supplementary Material**. Further inquiries can be directed to the corresponding author.

## Ethics Statement

The studies involving human participants were reviewed and approved by Mass General Brigham IRB Mass General Brigham 399 Revolution Drive, Suite 710 Somerville, MA 02145 Tel: 857-282-1900 Fax: 857-282-5693. Written informed consent for participation was not required for this study in accordance with the national legislation and the institutional requirements.

## Author Contributions

ZX: performing experiments, data analysis, and manuscript preparation. PJ: project designed and discussion, data analysis, and manuscript preparation. MM: data analysis and discussion. XJ: performing experiments, and data analysis. AF: project designed and discussion. CN: performing experiments, and data analysis. KL: performing experiments. YZ: project designed and discussion. KM: project designed and discussion, and data analysis. RS: project designed and discussion. XZ: project designed and discussion, performing experiments, data analysis, and manuscript preparation. All authors contributed to the article and approved the submitted version.

## Funding

This work was supported in part by the National Institutes of Health (R01 CA193520), the Jarislowsky Foundation, and a gift from Tom and Siobhan Quinn.

## Conflict of Interest

The authors declare that the research was conducted in the absence of any commercial or financial relationships that could be construed as a potential conflict of interest.

## Publisher’s Note

All claims expressed in this article are solely those of the authors and do not necessarily represent those of their affiliated organizations, or those of the publisher, the editors and the reviewers. Any product that may be evaluated in this article, or claim that may be made by its manufacturer, is not guaranteed or endorsed by the publisher.
